# Mechanical Dentistry

**Published:** 1883-08

**Authors:** J. Hooper


### Mechanical Dentistry.

BY DR. J. HOOPER.

[Read before the Kentucky State Dental Society.]

The Executive Committee having appointed me to discuss or have a paper on the subject of Mechanical Dentistry (although I have had but limited notice), I will endeavor to express a few practical thoughts.

I have heard some dentists disparage this branch of dental practice, but I deem it one of vast importance. I think it requires talent, thorough cultivation, and artistic ability, combined with thought and good judgment, to comprehend it and insure it a success.

The mechanical dentist must correct irregularities, he must possess the knowledge of the extent to which the arch should be expanded when contracted, when and how to remove teeth, when irregular in children, that the arch may develop as nature requires; not to extend the teeth, producing a narrow, contracted arch in mature life similar to that of a child of ten years. Suppose the six year molars be extracted; they occupy the space of half an inch on each side of the mouth; that space will contract, as those men who advocate the extraction of the six year molars will admit. A child having three molars extracted will frequently at the age of twenty years present a mouth in a badly-deformed condition, for this reason, from its teeth developed into a wide, round, normal arch the space of an inch in each arch has been permitted to contract, producing the deformity.

This is what is done, gentlemen, when the six year molars are extracted before the jaw is fully developed. I must take issue with you who advocate early extraction of the six year molars, for I have seen adult mouths deformed from having the lateral incisors and canines extracted for irregularities, also from the teeth being filed too much, especially when the lower arch is a little prominent. The arch will contract and let the upper fall in behind the lower teeth. I will prove my assertion by this model. You can see where the first bicuspids were extracted nine months ago by a dentist who has the practice and the confidence of some of the best citizens of this community. You can observe the deformity produced by such a course. In the fitting in of artificial teeth the best judgment and artistic skill is requisite.

The mechanical dentist must restore nature by replacing the features of the face and the expression lost by the loss of the teeth. He should be able to inform those desiring artificial teeth what restoration could be made for lost features and expression. He should know what teeth to select to correct deformity, and be able to inform what teeth could be treated and filled, and have the courage to refuse to extract teeth that might be saved.

One desirous of becoming a fine mechanical dentist should first learn operative dentistry, familiarizing himself with the norma position of the teeth, and study to treat and save them. Many learn dentistry backwards, by studying mechanical first. It is not surprising, therefore, to hear some clamoring for a divorce of operative and mechanical dentistry. I deem it a lack of ability on the part of all such. That is one reason why young men entering the profession say, after practicing awhile, "I don't like mechanical dentistry, but I do like the operative; I wish I could dispense with the former." Gentlemen, that proves to my mind that mechanical dentistry is the most difficult. It is a self-evident fact that if mechanical dentistry were placed in its proper sphere, and the public educated to what scientific mechanical dentistry is, we would fail to observe on our thoroughfares the deformed creatures it is now our lot to encounter, and we would, in a measure, rid our noble profession of its frauds.

